# Comparative effectiveness of an economic empowerment program on adolescent economic assets, education and health in a humanitarian setting

**DOI:** 10.1186/s12889-020-8219-6

**Published:** 2020-02-04

**Authors:** Nancy Glass, Mitima Mpanano Remy, Larissa Jennings Mayo-Wilson, Anjalee Kohli, Marni Sommer, Rachael Turner, Nancy Perrin

**Affiliations:** 1 School of Nursing, Johns Hopkins University, Baltimore, Maryland USA; 2 Programme d’Appui aux Initiatives Economiques (PAIDEK), Bukavu, Democratic Republic of Congo; 3 School of Public Health, Johns Hopkins University, Baltimore, Maryland USA; 4 School of Public Health, Indiana University, Bloomington, Indiana USA; 5 Institute of Reproductive Health, Georgetown University, Washington, District of Columbia USA; 6 Mailman School of Public Health, Columbia University, New York, New York USA

**Keywords:** Young adolescents, Economic empowerment, Humanitarian settings, Conflict, Health

## Abstract

**Background:**

Adolescence is a critical period of human development, however, limited research on programs to improve health and well-being among younger adolescents living in conflict-affected and humanitarian settings exists. The purpose of this study was to assess the comparative effectiveness of an economic empowerment program on young adolescent outcomes in a complex humanitarian setting.

**Methods:**

This longitudinal, mixed methods study examined the relative effectiveness of an integrated parent (Pigs for Peace, PFP) and young adolescent (Rabbits for Resilience, RFR) animal microfinance/asset transfer program (RFR + PFP) on adolescent outcomes of asset building, school attendance, mental health, experienced stigma, and food security compared to RFR only and PFP only over 24 months. A sub-sample of young adolescents completed in-depth qualitative interviews on the benefits and challenges of participating in RFR.

**Results:**

Five hundred forty-two young adolescents (10–15 years) participated in three groups: RFR + PFP (*N* = 178), RFR only (*N* = 187), PFP only (*N* = 177). 501 (92.4%) completed baseline surveys, with 81.7% (*n* = 442) retention at endline. The group by time interaction (24 months) was significant for adolescent asset building (X^2^ = 16.54, *p* = .002), school attendance (X^2^ = 12.33, *p* = .015), and prosocial behavior (X^2^ = 10.56, *p* = .032). RFR + PFP (ES = 0.31, ES = 0.38) and RFR only (ES-0.39, ES = 0.14) adolescents had greater improvement in asset building and prosocial behavior compared to PFP only, respectively. The odds of missing two or more days of school in the past month were 78.4% lower in RFR only and 45.1% lower in RFR + PFP compared to PFP only. No differences between groups in change over time were found for internalizing behaviors, experienced stigma, or food security. Differences by age and gender were observed in asset building, prosocial behavior, school attendance, experienced stigma, and food security. The voices of young adolescents identified the benefits of the RFR program through their ability to pay for school fees, help their families meet basic needs, and the respect they gained from family and community. Challenges included death of rabbits and potential conflict within the household on how to use the rabbit asset.

**Conclusion:**

These findings underscore the potential for integrating economic empowerment programs with both parents and young adolescents to improve economic, educational, and health outcomes for young adolescents growing up in rural and complex humanitarian settings.

**Trial registration:**

NCT02008695. Retrospectively registered 11 December 2013.

## Background

Adolescents represent about one-fourth of the world’s population [1], with almost 90% living in low- and middle-income countries (LMIC), however, most research on programs to improve health and well-being during adolescence has taken place in high-income countries [2] and with older adolescents (ages 15–19) [3]. Comparatively little attention has been paid to young adolescents (10–14 years) living in low-resource and humanitarian settings, although this is beginning to change with global efforts to build knowledge and evidence for interventions in diverse LMICs specific to the needs of younger adolescents with the recognition that this is a time of significant and rapid physical, emotional, social, and cognitive changes in their lives [4].

In eastern Democratic Republic of Congo (DRC), the setting for the study, rural populations of South Kivu province have experienced more than two decades of conflict, political instability, and extreme poverty [5–7]. Despite its vast physical size and abundant natural resources, the DRC is one of the poorest countries in the world, ranking 176th out of 189 countries and territories in the 2018 Human Development Index [6]. The situation in Eastern DRC remains volatile, with over 70 armed groups active in North and South Kivu provinces. In 2016 alone, the United Nations Children’s Fund (UNICEF)-coordinated Rapid Response to Movements of Populations (RRMP) program provided multi-sectoral (e.g., health, education, protection, housing) assistance to 2.3 million conflict-affected people. Child poverty is widespread and particularly concentrated in conflict-affected and hard-to-reach rural areas in Eastern DRC. According to UNICEF, 80% of DRC children under 15 years old have experienced at least two major deprivations (e.g., absences of food, housing, water, and medical care) during their childhood [8].

Practitioners and researchers have found that armed conflict directly (e.g., injuries, displacement) and indirectly (e.g., lack of educational opportunities, marital conflict due to economic insecurity) have negative effects on adult and child resilience (i.e., an ability to recover from or adjust to misfortune or change). Although children demonstrate resilience in the face of multiple adversities associated with conflict [9–11], evidence indicates an enabling environment that includes supportive adult family members and economic opportunities to limit the negative effects of adverse childhood events (ACEs) and prioritizes the health, education, and safety of children living in situations of ongoing stress is key to resilience [12]. Parents in humanitarian settings have identified the ongoing challenges of daily economic stress, marital conflict, and poor mental health as ways that compromise their ability to parent and can result in negative health, behavior, and educational outcomes for children [12–14]. To illustrate this, a study in rural villages experiencing deprivation and trauma associated with the prolonged conflict in DRC found that parent’s report of poor mental health and victimization or perpetration of intimate partner violence (IPV) had a negative impact on their young adolescents’ well-being, with different impacts for girls and boys. Specifically, parent’s symptoms of post-traumatic stress disorder (PTSD) and depression had a stronger negative effect on girls, including experienced stigma (i.e., called names or insulted), internalizing behaviors (i.e., symptoms of depression), and school attendance (missed two or more days of school in past month) than boys [15]. These differences in health and educational outcomes for girls and boys are related to multiple factors including gender norms (e.g., informal rules and shared social expectations) that are defined and enacted during adolescence [16–18].

Parents and young adolescents are vulnerable to multiple and inter-related poor health, economic, and social outcomes related to direct and indirect exposures to prolonged conflict. To reduce negative outcomes and build on the resilience of adults and young adolescents by creating an enabling environment, two economic empowerment programs were developed and implemented with parents and female and male young adolescents in a partnership between the Johns Hopkins School of Nursing and a Congolese microfinance institute, Programme d’Appui aux Initiatives Economiques du Kivu (PAIDEK). The purpose of this paper is to present the findings from the comparative effectiveness trial that engaged both parents and young adolescents in economic empowerment to improve economic, educational, and health outcomes for young adolescents growing up in rural and complex humanitarian settings.

The economic empowerment program, Pigs for Peace (PFP), a hybrid livestock microfinance/productive asset transfer has been published elsewhere but a review of the program follows [19]. PFP works with local authorities, leaders and residents in rural villages in South Kivu province of Eastern DRC to build sustainable livelihoods and social capital using traditional forms of assets (i.e., entities that hold economic value and can be converted to cash in times of opportunity or crisis. Farming and breeding animals (e.g., cows, goats, pigs, rabbits, chickens) are primary sources of assets and status for rural households. Pigs were selected as the asset for the program as they are common to the area, productive (e.g., having on average 6 piglets per gestation in our program), eat local food products and do not need large amounts of space to live and forage. Further, there are no religious (e.g., primarily Catholic and Protestant religions) or gender taboos (e.g,. women can own and sell pigs as they're not tied to the dowry system like cows/goats) about the breeding and selling of the family pig. Village households that are interested in participating in the program must complete the village-based group training provided by our skilled PFP implementation team prior to receiving the pig asset loan (e.g., female 2–4 month old piglet). The training focuses on: 1) raising and breeding healthy pigs; 2) participant’s commitment to building a pigpen and compost for pig waste to fertilize crops per project standards; and 3) repaying pig asset loan by reimbursing the project with two female piglets when their pig asset loan gives birth: one piglet to repay the loan and one piglet for interest on the loan. The PFP team commits to: 1) providing a vaccinated and healthy pig asset loan to each participating household; 2) ongoing support to participating households through monthly village based group meetings and home visitations to help participants problem solve challenges with pig health, care and breeding; and 3) access to a veterinarian technician as needed. Once the participating household reimburses the project with the two female piglets, these repayment piglets are then provided as an asset loan to waitlist PFP members often in the same community, with the focus of building social capital within the village. The original pig asset loan and the remaining piglets are for the household to continue to raise, breed, and sell with continued support of the PFP team as long as the household participates in the PFP program. A piglet (3 months of age) can be sold at $20–$25 in the area markets, a significant supplement to the estimated gross national income of US$795 annually [20].

The PFP effectiveness trial, published elsewhere [7], was implemented with 832 households in 10 rural villages. The findings supported the implementation of the PFP program in rural and conflict-affected settings where families have extremely limited access to employment or asset building opportunities, health, or social services, and where social norms that sustain gender inequality are strong. Following the success of the PFP program and at the request of parents active in the program, an adapted program was developed to be implemented with young adolescents (10–15 years) in rural households. The adapted program was named Rabbits for Resilience (RFR) and as the name implies, rabbits were provided as the productive asset loan to female and male adolescents with permission and support of parents/caregivers. The adolescents with a parent/caregiver completed a similar village based group training. The training focuses on: 1) raising and breeding healthy rabbits; 2) participant’s commitment to building a rabbit hutch and compost for rabbit waste per project standards; and 3) repaying rabbit asset loan by reimbursing the project with two rabbits when their rabbit asset loan gives birth: one rabbit to repay the loan and one rabbit for interest on the loan. The RFR implementation team commits to: 1) providing a vaccinated and healthy male and female rabbit asset loan to each participating adolescent; 2) ongoing support to participating youth and their parents through monthly village based group meetings and home visitations to help participants problem solve challenges with rabbit health, care and breeding; and 3) access to a veterinarian technician as needed. Once the participating adolescent reimburses the project with the two rabbits, these repayment rabbits are then provided as an asset loan to waitlist RFR members (e.g., siblings, friends, neighbors) often in the same community, with the focus of building peer relationships within the village. As with PFP, once the asset loan is reimbursed, the original rabbit asset loan and the remaining rabbits are for the household to continue to raise, breed, and sell with continued support of the RFR team as long as the adolescents are active in the RFR program. A rabbit (2 months of age) can be sold at $8–10 in the area markets and funds can be used by the girls and boys in the program to pay school fees and contribute to household and personal needs (e.g., food, clothes, shoes and medicine).

## Methods

### Study design and setting

The study compared the relative effectiveness of integrating RFR with PFP (RFR + PFP) in rural households on adolescent outcomes of asset building, school attendance, mental health, experienced stigma, and food security to: 1) households with adolescent participants in RFR only and 2) households with parent participants in PFP only (i.e., no adolescents in the household were in RFR). The study measured the outcomes over a 24-month period and was conducted in the same 10 villages as the PFP effectiveness trial [7]. In addition, a purposive sub-sample of male and female young adolescents participated in in-depth qualitative interviews (at approximately 12 months post baseline) with trained staff on the perceived benefits and challenges of RFR participation. Adolescents were specifically asked to discuss their experience in raising the rabbits, what supports or challenges they may have encountered, how they used any earnings from selling rabbits, and how they perceived RFR affected their status in their community and families. The 10 rural villages for the study were selected based on: (1) feasibility of conducting and managing the study and intervention over a diverse geographic area; (2) village leadership commitment to PFP, RFR, and the study; and (3) existing relationships between the partner Congolese organization and village leadership.

### Participant recruitment and eligibility criteria

**Fig. 1:**
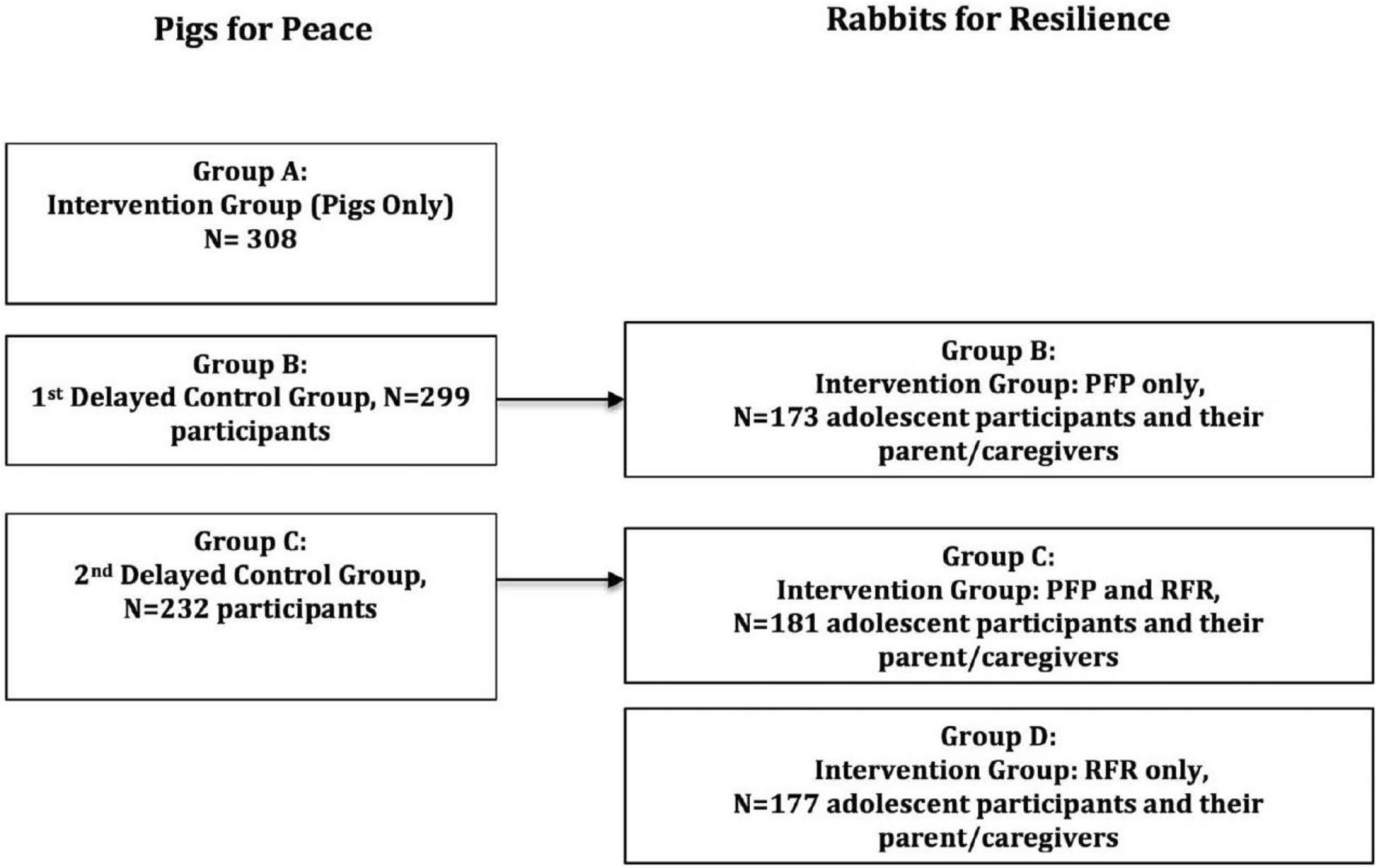
Study design

Community meetings were held first with parents participating in the established PFP program and other adults in the community interested in PFP. The meetings provided details on the RFR and PFP programs and the associated study. The PFP effectiveness study enrolled households with men and/or women (16 years and older) who expressed an understanding and commitment to productive asset/microfinance programs (e.g., credit and repayment of loans), were permanent residents of the village, and were responsible for the household. The eligibility criteria of 16 years and older accommodated rural realities where, due to the conflict, elder siblings are caregivers for younger family members and some women and men are married and have children by 16 years of age. Details on the PFP recruitment/eligibility process are published elsewhere [7]. Male and female adolescents aged 10–15 years of age were eligible for the study if: (1) their parent/guardian was enrolled in the PFP program, but was in the delayed control group and had not yet received the pig asset loan; (2) youth and their parent/guardian expressed an interest and commitment to the program (e.g., willing to build a rabbit hutch, attend meetings, repayment of rabbit offspring and transfer of asset (rabbit) to other adolescent participants); and (3) were permanent residents of the participating village. We expanded the eligibility age to 15 years for the study at the request of parents and our study partner. Although young adolescents are typically defined as age 10–14 years, parents felt 15 years was a vulnerable age and that engaging them in RFR may prevent them from leaving the village to find work in the mines or marrying early. Parents/guardians provided consent for their eligible child to participate in RFR and the study prior to children being approached for assent. As the RFR study builds off of the sample of households randomized into the PFP trial, randomization of youth was not conducted for RFR. An eligible male or female adolescent in each participating PFP household was selected. If the household had more than one eligible adolescent, the parents selected the adolescent to participate (see Fig. 1). Parents with eligible adolescents in the household enrolled in the first PFP delayed control group comprised the PFP only group, which did not include a loan of a rabbit to the youth during the study period. Parents with eligible adolescents enrolled in the second PFP delayed control group comprised the PFP + RFR group. Parents for the RFR only group were recruited through outreach to village households with the help of local leaders to identify eligible and interested adults and young adolescents. Households that were recruited for RFR only had not previously participated in the microfinance/asset transfer program.

### RFR microfinance/asset transfer program

RFR works with young male and female adolescents (10–15 years of age) that are interested and committed to raising rabbits, including building a rabbit cage, providing care for the rabbit, participating in a training program and meetings with support by our skilled RFR implementation team, and repaying the loan to the project in the form of two female rabbits when the original rabbit gives birth. These rabbit loan repayments are then given to other adolescents in the project but not in the study. Similar to PFP, the original rabbit loan and remaining offspring are for the RFR members to continue to raise, breed, eat, and sell as decided by the adolescent in collaboration with their parent/caregiver and mentorship from our team. While continuing to breed and raise rabbits, the adolescents have an option to sell some of their rabbits at local market rates (approx. 10 USD value) to pay for school fees, purchase food or medicine, or invest in other income generating activities. The RFR + PFP implementation team facilitated meetings and home visits with parents in PFP and adolescent members in RFR to discuss challenges and identify solutions related to participation, guide members in raising livestock/animals, and encourage timely repayment of pig and rabbit loans. Our team also facilitated regular visits by a project veterinarian technician to review animal care protocols and provide vaccinations as appropriate and needed.

### Study outcomes

The main outcomes of this study focused on young adolescent asset building, school attendance, mental health, experienced stigma, and food security. The study survey was developed after a review of existing and validated tools that have been used with young adolescents in similar low-resource and conflict-affected settings and through multiple field tests with adolescents not living in study villages. Survey refinement was an iterative process that lasted several months and included translation and back translation from English to French and from French to local languages (Swahili, Mashi) for administration by Congolese team members trained to conduct interviews with young adolescents.

### Asset building

The asset building measure was developed for the study to examine young adolescent perceived resources that will move them towards economic well-being now and in the future. The measure consisted of six items (e.g., I am learning how to better earn cash or acquire income generating assets for myself and family, I have more ways now of financially supporting myself and family than I did 1 year ago, I am acquiring the skills I need to earn the income I desire). Participants responded yes or no to each item. The score is the proportion of items endorsed. Cronbach’s alpha = 0.81 in this sample.

### School attendance

Adolescents enrolled in school answered questions about the number of full days of school that they missed in the past one-month. The number of days missed was measured on a 1–4 scale (0, 1–2 days, 3–5 days, and 6 or more days missed from school in the past month). Since the distribution was extremely skewed, this variable was dichotomized to adolescent report that he/she missed two or more days in the last month versus missed 0–1 days in the last month. Participants that were not enrolled in school were assigned a value of 1 (equivalent to missed 2 or more days of school in the last month).

### Adolescent mental health

The reduced Acholi Psychosocial Assessment Instrument (APAI) was developed for use with young adolescents living in rural, post-conflict Northern Uganda [21, 22]. The Internalizing and Prosocial subscales of the APAI were used to assess mental health outcomes. Items are assessed on a 4-point response scale (i.e., never, sometimes, often, always) over the past 7 days. The 19-item Internalizing subscale reflects anxiety and depression (e.g., has constant worries, thinks she/he is of no use, cries when alone) and had a Cronbach’s alpha = 0.70 in this sample. The 8-item Prosocial subscale reflects positive social behaviors (e.g., listens to others and elders, plays together with others, helps others) and had a Cronbach’s alpha = 0.84 in this sample.

### Experienced stigma

We adapted a measure of everyday discrimination [23, 24] to look at “chronic, routine and relatively minor experiences of unfair discrimination” [24] or stigma experienced by adolescent participants. Participants answered eight questions (e.g., people act as if they are afraid of you, people treat you with less respect than others, you are called names or insulted) on a 3-point scale (i.e., never, sometimes, always) about the frequency of different types of experienced stigma occurring in their day-to-day life. Cronbach’s alpha in this sample was 0.79.

### Food security

The Household Dietary Diversity Scale (HDDS) [25] assessed the total number of food groups (range: 0–12 items) consumed by the household members in the previous day and night as reported by the adolescent. Food security as measured by the HDDS is also “used as a proxy measure of the socio-economic level of the household” [25].

### Data collection

The baseline survey with young adolescents took place after recruitment but prior to RFR program training that included details on health, nutrition, and well-being of the rabbit, building the hutch, composting waste, asset loan distribution, and repayment. Assenting adolescents were informed at the beginning of the survey and during the discussion of sensitive topics (e.g., mental health) and that they could stop or refuse to answer or skip questions at any point without consequence to their participation in RFR. Surveys were conducted after the school day was complete or on the weekend in a private place (i.e., in home or outside the home) identified with the adolescent. Trained Congolese staff used a tablet with the pre-programmed study survey. Use of the tablet was successful in our previous PFP trial in multiple ways: (1) reduced logistical burden of printing and managing the paper questionnaires; and (2) ensured real-time access to the data to monitor data quality and identification of issues so that they could be remedied between surveys. Additionally, participants expressed confidence and comfort when answering questions with the use of the tablet as compared to a paper-based survey where staff write down responses. Adolescents that completed the survey received approximately 1.50 USD for their time (45–90 min), an amount recommended by study partners and consistent with previous studies. Data recorded on tablets are encrypted. Once uploaded to a central US-based server, the data are automatically erased from the tablet. Follow-up interviews were conducted with adolescents at 12 and 24-months post-baseline.

In addition to the surveys, a purposive sub-sample of 30 young male and female adolescents across age ranges in both the RFR only and RFR + PFP groups in each village were selected by the team and invited to participate in in-depth qualitative interviews. Each team member selected three young adolescents that had been active in RFR as well as those that appeared to struggle in the program to learn about the benefits and challenges of participating in RFR and their recommendations for revisions to the programs. We strived to select and recruit equal numbers of girls and boys stratified by age. The in-depth qualitative interviews were conducted at about 12 months from baseline interviews. The in-depth interviews were conducted in private after parental consent and adolescent assent, and lasted on average for 60 min. Participants were reimbursed approximately 1.50 USD for their time.

Study identification codes and names were recorded during one-on-one surveys and in-depth interviews. All data recorded through the tablet-based program and audio recorder were uploaded to a password-protected server managed by the study team. Names were centrally removed and stored in a separate file.

### Ethics approval

The Johns Hopkins Medical Institute (JHMI) Institutional Review Board (IRB) approved this protocol. As at the time there was no local IRB functioning in South Kivu province of Eastern DRC, an ad hoc committee of respected Congolese scholars at the Université Catholique de Bukavu and community members reviewed the research and intervention protocols before giving approval for the study. A letter of approval from the Congolese scholars was submitted to the Hopkins IRB. With approval from the local experts and Hopkins IRB, all surveys and in-depth interviews were conducted after a parent/guardian provided informed oral consent for their child to participate and the adolescent provided voluntary, informed oral assent to our skilled research team members. Oral consent was chosen as the majority of parents/guardians (60%) had never been to school and were unable to write their name or the name(s) of their children. Oral consent and assent allowed for no names to be linked to survey or in-depth interview data.

### Sample size and power

Power for the study is based on a sample size of 480 young adolescents (160 per group), power of 0.80, and α level of 0.05. The study can detect a significant difference between groups if the change over time in APAI [21, 22] scores is 2.67, 2.82, and 2.98 greater in one group for ICCs of 0.001, 0.005, and 0.01, respectively.

### Statistical analyses

Differences between the three groups (RFR + PFP, RFR only, PFP only) on baseline characteristics were compared with generalized linear models with robust standard errors to account for the nesting of adolescents within the 10 villages. Three-level mixed models were used for the main analyses. Time (baseline, 12, and 24 months) was nested within adolescents and adolescents were nested within villages. Group, time, and the group by time interaction were included in the model. Normal Gaussian distribution models were used for all analyses except for school attendance (missing two or more days of school in past month) which used a logistic model. All adolescents (*N* = 542) were included in the analyses. Mixed models do not require complete data at all time points so all available data were included in the analyses. Analyses were intention-to-treat with adolescents in the group as assigned even if they did not receive a rabbit asset loan (e.g., were unable to build a suitable hutch for the rabbit). Exploratory stratified mixed models by age group (10–11, 12–13, and 14–15 years) and sex were conducted to determine if the differences between groups varied across age and sex. Since the study was not powered for these analyses, effect sizes were examined and compared across strata.

### Qualitative analyses

All qualitative interviews were recorded and transcribed by local team members from the local languages (Swahili or Mashi) into French. A code list was then developed by co-author LMJW to identify adolescent responses relating to benefits and challenges of participating in the RFR program by study outcomes (e.g., asset building, health, and school attendance). The code list was reviewed with Congolese team members for accuracy and applied to the French transcripts. All coded statements were then extracted, translated to English, and aggregated for analysis using qualitative descriptive methods. To understand the relative frequency of coded statements, a count of the number of interviews in which each theme was discussed was also tabulated. As a final step, a sample of statements from each of the emergent themes was selected for presentation to characterize the voice of the young adolescent participants in this article. All quoted statements were labeled by gender, age, and village.

## Results

A total of 542 young adolescents (10–15 years) participated in three groups: RFR + PFP (*N* = 178), RFR only (*N* = 187), and PFP only (*N* = 177). In the RFR + PFP group 165 (92.7%) young adolescents completed baseline, 152 (85.4%) completed 12-month, and 154 (86.5%) completed 24-month surveys. In the RFR only group 169 (90.4%) young adolescents completed baseline, 138 (73.8%) completed 12-month, and 142 (75.9%) completed 24-month surveys. In the PFP only group 167 (94.4%) young adolescents completed baseline, 129 (72.9%) completed 12-month, and 144 (81.4%) completed 24-month surveys. The three groups were not significantly different on adolescents’ age (*p* = .593), sex (*p* = .423), general health (*p* = .602), or number of trauma experiences in their lifetime (*p* = .257). Table 1 summarizes the baseline characteristics for the three groups.

**Table 1:** Baseline characteristics and outcomes by group

	PFP only *N* = 177	RFR + PFP *N* = 178	RFR only *N* = 187
Age (years)			
10	42 (23.7%)	50 (28.1%)	54 (28.9%)
11	17 (9.6%)	21 (11.8%)	18 (9.6%)
12	35 (19.8%)	29 (16.3%)	31 (16.6%)
13	36 (20.3%)	24 (13.5%)	34 (18.2%)
14	25 (14.1%)	23 (12.9%)	24 (12.8%)
15	22 (12.4%)	31 (17.5%)	25 (13.4%)
Female	99 (55.9%)	89 (50.0%)	96 (51.3%)
General Health Mean (SD)	3.05 (0.96)	3.14 (0.83)	3.08 (0.85)
Number of trauma experiences Mean (SD)	2.38 (1.99)	2.04 (1.92)	2.26 (2.25)
Outcome Measure at Baseline, Mean (SD)			
Experienced stigma	1.27 (0.32)	1.29 (0.33)	1.25 (0.33)
Internalizing Behavior	1.32 (0.24)	1.36 (0.28)	1.33 (0.27)
Prosocial Behavior	3.00 (0.59)	2.85 (0.55)	2.93 (0.57)
Asset Building	1.82 (2.08)	1.71 (1.87)	1.66 (1.88)
Food Security	3.43 (1.88)	3.00 (1.76)	3.08 (1.63)
School Attendance (missed 2 or more days in past month)	*n* = 39 (25.2%)	*n* = 45 (30.0%)	*n* = 60 (39.0%)

### Main analyses

The group by time interaction was significant for asset building (X^2^ = 16.54, *p* = .002), school attendance (X^2^ = 12.33, *p* = .015), and prosocial behavior (X^2^ = 10.56, *p* = .032). At 24 months, in groups with adolescents’ participation, both RFR + PFP and RFR only groups, the adolescents had greater improvement in asset building (RFR + PFP ES = 0.31; RFR only ES = 0.39) than the PFP only group. Compared to adolescents in the PFP only group, the RFR + PFP group (ES = 0.38) and RFR only group (ES = 0.14) adolescents had greater improvement in prosocial behavior, with the effect being greatest for adolescents in RFR + PFP. For school attendance, at 24 months, the odds of adolescents missing two or more days in the last month was 78.4% lower in the RFR only and 45.1% lower in the RFR + PFP group compared to the PFP only group. No differences between the groups in change over time were found for internalizing behaviors on the mental health measure, experienced stigma, or food security. Examining the effect sizes across the two comparisons, it can be seen that RFR + PFP had a greater impact than RFR only on prosocial behavior (ES = 0.38 vs. 0.14) and food security (ES = 0.28 vs. 016) and similar impact for asset building (ES = 0.31 vs. 0.39), while RFR only had a greater impact than RFR + PFP on school attendance (OR = 0.22 vs 0.55). Table 2 summaries the results of the main analyses.

**Table 2:** Main outcome means, standard deviation or percent with associated effect sizes (Cohen’s d’ or Odds Ratio)

	Experienced Stigma	Internalizing Behavior	Prosocial Behavior	Asset Building	Food Security	School Attendance^a^
PFP Only						
Baseline	1.27 (0.32)	1.32 (0.24)	3.00 (0.59)	0.30 (0.35)	3.43 (1.88)	25.16%
12 months	1.16 (0.21)	1.21 (0.21)	3.02 (0.54)	0.52 (0.34)	3.76 (1.58)	35.24%
24 months	1.16 (0.22)	1.21 (0.21)	3.00 (0.54)	0.52 (0.33)	3.76 (1.54)	31.36%
RFR Only						
Baseline	1.25 (0.32)	1.33 (0.27)	2.93 (0.57)	0.28 (0.32)	3.08 (1.63)	30.00%
12 months	1.17 (0.28)	1.24 (0.24)	3.02 (0.53)	0.62 (0.32)	3.72 (1.60)	30.47%
24 months	1.16 (0.28)	1.24 (0.24)	3.01 (0.52)	0.62 (0.33)	3.69 (1.61)	30.00%
RFR + PFP						
Baseline	1.29 (0.33)	1.36 (0.28)	2.85 (0.55)	0.28 (0.31)	3.00 (1.76)	38.96%
12 months	1.16 (0.25)	1.26 (0.25)	3.07 (0.55)	0.61 (0.31)	3.82 (1.61)	29.66%
24 months	1.16 (0.24)	1.25 (0.24)	3.09 (0.55)	0.60 (0.31)	3.82 (1.61)	28.46%
Group by time Interaction	χ^2^ = 2.22 *p* = .695	χ^2^ = 0.96 *p* = .915	χ^2^ = 10.56 *p* = .032	χ^2^ = 16.54 *p* = .002	χ^2^ = 6.27 *p* = .180	χ^2^ = 12.33 *p* = .015
Effect size PFP only vs. RFR only	d’ = 0.08	d’ = 0.04	d’ = 0.14	d’ = 0.39	d’ = 0.16	OR = .216
Effect size PFP only vs. RFR + PFP	d’ = − 0.05	d’ = − 0.03	d’ = 0.38	d’ = 0.31	d’ = 0.28	OR = .549

^a^Percent who missed 2 or more days in past month

### Analyses stratified by age

**Fig. 2:**
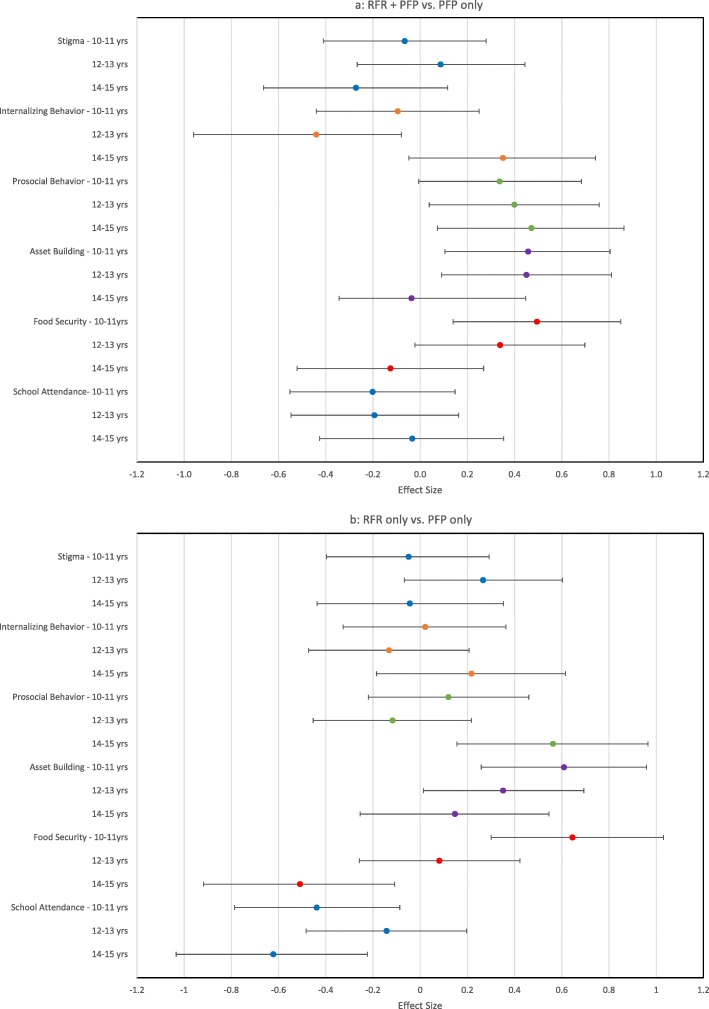
(**a**) Effect sizes for RFR + PFP versus PFP only and (**b**) RFR only versus PFP only stratified age

The effect of the intervention varied by age. Figures 2a and b summarize the relative effect sizes for the main outcomes stratified by age. Asset building improved for the youngest age group (10–11 years) for both the RFR + PFP (ES = 0.46) and RFR only (ES = 0.61) groups in comparison to the PFP only group. The same effect was seen for adolescents in the middle age range (12–13 years) for RFR + PFP (ES = 0.45) and RFR only (ES = 0.35) groups in comparison to PFP only. The effect was smaller for the older age group (14–15 years), however, the older adolescents had higher asset building (M = 2.35, SD = 2.09) than younger age groups (12–13 years, M = 1.67, SD = 1.83; 10–11 years, M = 1.31, SD = 1.82) at baseline. Relative to the adolescents in the PFP only group, improvement in prosocial behavior for the RFR + PFP group was consistent across the three age groups (10–11 years ES = 0.34; 12–13 years ES = 0.40; 14–15 years ES = 0.47). Adolescents age 14–15 years in the RFR only (ES = 0.56) group showed improvement in prosocial behavior compared to adolescents of the same age in the PFP only group. Food security improved relative to the PFP only group for the youngest adolescents (10–11 years) in RFR + PFP (ES = 0.49) and RFR only (ES = 0.65) groups, as well as for adolescents age 12–13 years in the RFR + PFP group (ES = 0.34). Across groups, adolescents in the oldest age group (14–15 years) were more likely to miss two or more days of school in the past month (37.9%) at baseline, followed by adolescents 10–11 years (26.9%) and 12–13 years (21.0%). Adolescents age 14–15 years in the RFR only group showed the most improvement in school attendance (ES = -.62) compared to PFP only, followed by the youngest age group (10–11 years, ES = -.44) and middle age group (12–13 years, ES = -.14). Among the 12–13 age group, a reduction in internalizing behavior was noted for RFR + PFP (ES = − 0.44) compared to the PFP only group. No effects for experienced stigma were found for any of the age groups.

### Analyses stratified by gender

**Fig. 3:**
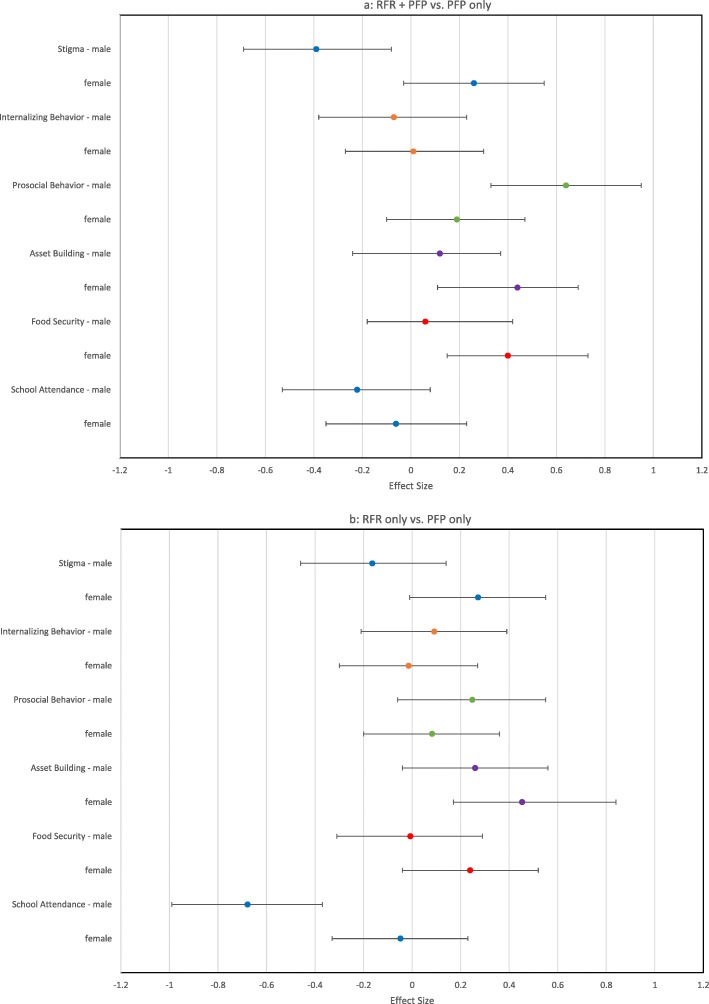
(**a**) Effect sizes for RFR + PFP versus PFP only and (**b**) RFR only versus PFP only stratified gender

Figures 3a and b summarize the relative effect sizes for the main outcome variables stratified by sex. Female adolescents in the RFR + PFP (ES = 0.44) and RFR only (ES = 0.45) groups had an increase in asset building compared to the PFP only group. Smaller impacts on asset building were observed for male adolescents (ES = .12 for RFR + PFP and ES = .26 RFR only). Male adolescents in the RFR + PFP had a reduction in experienced stigma (ES = − 0.39) and an increase in prosocial behavior (ES = 0.64) compared to PFP only, whereas females did not experience a similar impact on experienced stigma (ES = .26) and prosocial behavior (ES = 19). Across groups at baseline, male adolescents (33.8%) were more likely to report missing two or more days from school in the past month than females (29.3%). When adolescents in the RFR only group were compared to adolescents in the PFP only group, the reduction in days missed from school in the past month was stronger for male adolescents (ES = -.68) than females (ES = -.05). Female adolescents in RFR + PFP had an increase in food security (ES = .40) compared to the PFP only group and male adolescents (ES = 0.23).

### Analyses of qualitative interviews

Thirty-two qualitative interviews with adolescents, aged 11 to 16 (i.e., participant was 15 years of age when started in RFR) were conducted. Fifty-nine percent (*n* = 19) of interviews were with female adolescents. Eight themes emerged from the interviews which were categorized into three domains regarding adolescents’ perceived impact of RFR (e.g., economic, psychosocial, and health impacts). Selected quotations describing each theme are listed in Table 3.

**Table 3:** Summary of emergent themes with example quotations from qualitative interviews with adolescents

Inquiry Domain	Emergent Themes [With Relative Frequency^a^]	Example Quotations *(Translated to English from French)*
Economic	Using rabbit sales to pay for school fees and other school items^e^	• “*I was very happy because … I was able to attend school and pay for my studies … That I take good care of this rabbit because it can pay for my schooling and feed me.”* • *“In terms of finances, they pay for my school easily. No longer do they say ‘you didn’t pay yet, go home!’“* • “*I sold them and paid for my schooling. I paid for two months at school*.” • “*With the first birth, the rabbit birthed four bunnies and I sold the four for eight thousand fc and I had paid for two months at school, and that really helped. And the second birth, she had three bunnies. One died, the other two lived. And the two that lived I had them reimbursed.”* • “*I had sold two to pay for my brother’s schooling*.” • “*I buy clothes to wear to school and notebooks, a backpack and I bought shoes*.” • *“I was happy that the rabbit could give birth and I could sell the bunnies to pay for school, buy clothes, or oil from the village.”* • *“The 2nd time, I sold it and paid for school for our kids and for me as well. The 3rd birth, I did the same thing.”* • *“This [rabbit] can help me pay for school and buy food and clothes... I had sold the bunnies and paid this money... I was the first to pay the money for TENAFER (Test Nationale de Fin d’Etude Primaire, National Examine at the End of Primary School).”*
	Challenges sustaining rabbit business^b^	• *“For the first time, they were all eaten. The rats. Yes, they had entered into the rabbit’s room. Yes, I closed it off well, but they were too small.”* • *“It was hard to find grass. There were times where someone was missing grass and searching is difficult.”* • *“When we don’t have vegetables, they can ask me to butcher it, and if I refuse that can bring conflict.”* • *“The difficulty I had … [was when] the rabbit would escape too often and taught its little ones to escape. Since we sold it, it’s babies escaped too and go wandering.”* • *“Only one of the twelve bunnies grew, and the others started dying. And, just this little one, the thieves broke in and took it.”*
Psycho-social	Helping parents or others in the family and community^c^	• *“I can now pay for school costs, buy notebooks, and buy the uniform. [I feel] very, very good because my parents didn’t have the money to pay for my schooling. [I feel] very, very good because my parents didn’t have the money, and I helped.”* • *“For me, I find that I can help the others. I can give them a rabbit.”* • *“By selling several of the rabbits … after the sale, we gave one to someone else who has nothing.”* • *“This money, if someone gets sick, your friend, you can give it to him to go see the doctor, and he’ll get better with it.”* • *“I did well... I had bought shoes and clothes and payed for school for my little one.”*
	Improving respect given to adolescent from the community^c^	• “*The rabbit can change a man’s life. When we sell them and we have money, it changes one’s life. Money, when we see you financially well off because of the rabbit, you gain respect*.” • *“I was happy because I also discovered animal husbandry, and the others will never ignore me.”* • “*When you have money, you’re going to take good care of yourself, and they will say that his farming techniques improved, whereas before he had this rabbit and it wasn’t this way so the rabbit can earn you respect. If she births four, I’ll definitely give Mom one. And, when that one gives birth, she will give some to the other family members, and we really see the importance.”* • *“When I sell it, and there is money it pays for my school, and I am respected. Because when they see it, they like it too.”* • *“They (my family) consider me very highly. That’s to say Mom thinks highly of me because I brought the bread, and me, it’s going to help me with school little by little.”* • *“It will bring me respect because I can sell it, and when I get lots of money that brings respect.”*
	Reducing idleness and risk behaviors^b^	• *“Before, I was wandering the neighborhood. The time I spent wandering, I now spend looking for grass for the rabbit.”* • *“Like, I already have the rabbit. I have to work every day so that he can eat.”* • *“Because it’s very useful. Like when at home, the kids play and fight and don’t realize the importance of the rabbit. I tell them to help me because I have a lot to do. And then, they can help me do certain chores, and I do the others.”* • *“It helps me with a lot of things, like sleeping without eating, sending me back to school, missing clothes, wandering in the street … Like going in the street because I don’t have anything to do because there is no work to do. Like going wandering somewhere, we don’t even know.”*
Health	Experience of or plans to use rabbit sales towards unexpected medical costs^c^	• *“Yes, I went to sell the bunnies to buy medication. My head hurt, … the headache.”* • *“Mama added money to her savings for the hospital visit... I did that.”* • *“My rabbit- I liked it a lot because you can sell it if you get sick and pay for the medicine.”* • *“The rabbit can help me find medication.”* • *“The day I get sick, it will help me pay the clinic.”* • *“Someone can get sick, and he can sell a rabbit to get treatment.”* • *“When we are suffering, we can buy medication and drink it.”*
	Feeling happier, and less worried^b^	• *“And me, I feel good and my heart changed because I am no longer the person I was before I made the cage because with my rabbit, I don’t worry anymore. All I have to do is pick herbs and give them and as soon as I want to sell, I can sell.”* • *“If I’m given the money, I feel good.”* • *“When I sold the rabbit for money, I was happy.”*
	Providing source of food or hunger reduction^d^	• *“There are many changes... many things changed because I couldn’t eat anything and [now] I have this rabbit. They can no longer chase me out of school. I no longer lack shoes, clothes. And, I help my parents with these rabbits.”* • *“If we don’t have food, I bring the flour... [and] buy clothes, shoes.”* • *“It helps at home with food, because we can no longer sleep without eating.”* • *“It can help me against hunger. And, if I get sick, I can sell it and get money to receive medical treatment. And if I am sick, we can butcher it and eat it if hungry.”*

Note: ^a^Relative frequency of responses are denoted by: ^b^mentioned in < 25% of IDIs; ^c^mentioned in 25 to 50% of IDIs; ^d^mentioned in 51 to 75% of IDIs; ^e^mentioned in ≥ 75% of IDI

In summary, the young adolescents noted the economic impact was primarily on their ability to use the proceeds from their rabbit sales to pay for school fees and other school items, and appeared to additionally benefit from participating in RFR by being able to help their parents with school fees, medicine, and reducing hunger in the household. As two participants said:

*“I can now pay for school costs, buy notebooks, and buy the uniform. [I feel] very, very good because my parents didn’t have the money to pay for my schooling. [I feel] very, very good because my parents didn’t have the money, and I helped.”* – Girl, Age 13–15.

*“There are many changes … many things changed because I couldn’t eat anything and [now] I have this rabbit. They can no longer chase me out of school. I no longer lack shoes, clothes. And, I help my parents with these rabbits.”* – Girl, Age 13–15.

*“It helps at home with food, because we can no longer sleep without eating.”* – Boy, Age 10–12.

Another adolescent explained the respect she and her family gained by giving and transferring rabbits to other family members. Adolescents use the word respect to demonstrate their increased status by being productive and contributing to the family. As two participants said:

*“When you have money, you’re going to take good care of yourself, and they will say that his techniques improved, whereas before he had this rabbit and it wasn’t this way so the rabbit can earn you respect. If she births four, I’ll definitely give Mom one. When that one gives birth, she will give some to the other family members, and we really see the importance.”* – Girl, Age 13–15.

*“They (my family) consider me very highly. That’s to say Mom thinks highly of me because I brought the bread, and me, it’s going to help me with school little by little.”* – Boy, Age 10–12.

In addition, RFR participants expressed feeling happier and less worried about themselves and their families as a result of raising and selling rabbits. Two adolescents stated:

*“And me, I feel good and my heart changed because I am no longer the person I was before I made the cage because with my rabbit, I don’t worry anymore. All I have to do is pick herbs and give them and as soon as I want to sell, I can sell.”* – Boy, Age 10–12.

*“I was very happy because … I was able to attend school and pay for my studies … That I take good care of this rabbit because it can pay for my schooling and feed me.”* – Girl, Age 10–12,

Given the prolonged conflict and loss of livelihood opportunities in rural villages, parents had frequently expressed concern to our PFP team about their children, that their idleness would lead to risky behaviors that would negatively impact their future. As stated above, at the request of parents, the RFR program aimed to help them provide productive activities for their children that taught skills and provided resources to stay in school and avoid trouble. We asked adolescents to describe the benefits of the program in this regard. Two young boys stated that:

*“It [RFR] helps me with a lot of things, like, sleeping without eating, sending me back to school, missing clothes, wandering in the street … Like going in the street because I don’t have anything to do because there is no work to do. Like going wandering somewhere, we don’t even know.”* – Boy, Age 10–12.

*“Because it’s very useful. Like when at home, the kids play and fight and don’t realize the importance of the rabbit. I tell them to help me because I have a lot to do. And then, they can help me do certain chores, and I do the others.”* – Boy, Age 13–15.

Participants also identified some challenges in RFR, including dealing with the loss of rabbits due to death and theft, or anticipating conflict in determining whether to use the rabbits for family meals or further investment in their project. Example quotations from adolescents were:

*“Only one of the twelve bunnies grew, and the others started dying. And, just this little one, the thieves broke in and took it.”* – Boy, Age 10–12.

“*When we don’t have vegetables, they can ask me to butcher it [rabbit], and if I refuse that can bring conflict.”* – Boy, Age 13–15.

*“For the first time, they were all eaten. The rats. Yes, they had entered into the rabbit’s room. Yes, I closed it off well, but they were too small. The second time, she had five. Four died, and I sold one.”* – Boy, Age 13–15.

## Discussion

This study provides important contributions to our knowledge on asset building, health, and education outcomes for young adolescents that participate in economic empowerment activities in a humanitarian setting. The findings demonstrate that not only does the hybrid microfinance/productive assets loan program provide economic empowerment for the young adolescent and family, but also improves adolescent health and school attendance in a rural humanitarian setting. This finding is consistent with other evidence that demonstrated benefits beyond economics with asset-based economic interventions such as improved psychosocial outcomes [26, 27]. Tangible assets, such as the rabbit and offspring, can influence the adolescent’s future outlook and reduce risk behaviors such as leaving school early to work in the local mines. The findings also reinforce the role of parents/guardians in supporting productive activities for adolescents as they transition into adulthood [28]. Specifically, we found that asset building for the adolescents in RFR was strengthened when their parents were also involved in our evidence-based adult microfinance/asset transfer program, PFP.

The youngest age groups (ages 10–11 and 12–13) and female adolescents that participated in either the RFR + PFP or RFR only groups had greater improvement in asset building than the older age group (14–15 years) and male adolescents. This may be related to increased opportunities for young girls to engage in activities outside the household that are considered safe and acceptable by parents and the wider community. For example, the young female and male adolescents joined together with our RFR team at the local church or other village settings to discuss the program, their progress, and learn from older adolescents in the program. Additionally, the young girls were given the opportunity to join their peers and older siblings to go to the market to sell their rabbit(s) and contribute funds to their education and other family needs. These empowerment opportunities that support girls from young ages (10–13) in productive activities outside the home can help to challenge restrictive gender norms that minimize the potential of young girls to contribute to meet the needs of the family. Further, the young girls when meeting with the other members of the program in their village had opportunities for positive interactions with older adolescents and mentors, such as the RFR team. This finding is important given that gendered vulnerabilities combined with the financial instability frequently experienced in humanitarian settings can increase adolescent girls’ likelihood of experiencing sexual violence and coercion. Research has demonstrated the salience of gender inequitable power dynamics in conflict-affected settings, especially as it relates to forced or coerced early marriage or sexual activity [29]. Further, in protracted humanitarian settings, such as DRC, in addition to economic empowerment, an enabling environment where girls and boys receive support and mentorship from older siblings and peers, parents, and other adults can act as an important buffer against the multiple vulnerabilities created by growing up in an extremely challenged setting [30].

Young adolescents that participated in either RFR + PFP or RFR only groups had significant improvement in the prosocial behavior subscale of the APAI compared to the PFP only group. However, the greatest improvement in prosocial behaviors came for adolescents across all age groups in the RFR + PFP group. This is an important finding given that our previous research, as well as others, has demonstrated the potentially negative effects parents’ poor mental health and marital violence has on adolescent health and social outcomes [15, 29]. Engaging both parents and young adolescents in economic empowerment programs can result in the sharing of workloads to care for the animals (e.g., building the pen/hutch, preparing food for both the pig and rabbits, discussing use of proceeds from selling the animals) and strengthening the relationship not only between the adolescent and the parent, but also with other members of the family. Male adolescents in the RFR + PFP group were more likely than female adolescents to report a reduction in experienced stigma and an improvement in prosocial behavior. This may be related to family and community members identifying the positive change they observed in the behavior of boys in the program and the change the participants noted in qualitative interviews of being respected and able to contribute to the family’s well-being. The change in prosocial behavior was greatest among adolescent boys in the older age group (ages 14–15), an important age for risk taking behaviors such as alcohol use, unprotected sexual activities, and exposure to violence.

For school attendance, adolescents in RFR only and RFR + PFP groups were significantly less likely to miss two or more full days of school in the past month than adolescents in the PFP only group, with RFR only adolescents having the greatest improvement in school attendance. Adolescents most frequently discussed in the qualitative interviews using the funds from selling rabbits to pay for school or school-related expenses. Older adolescents, both girls and boys ages 14–15 years and boys in in the RFR only group, also showed the most improvement in school attendance. Providing adolescents opportunities to generate income through activities within their village to stay in school reduces the need for adolescents, especially boys, to leave their home and community prior to completing secondary school to find livelihood opportunities that often involve working in dangerous settings such as mines. More than 60% of the world’s supply of cobalt is mined in the “copper belt” of the south-eastern provinces of DRC, the setting for the study. Cobalt is found in every lithium-ion rechargeable battery in smartphones, tablets, computers, and electric vehicles, so the demand is immense. Adolescent males and females go to the cobalt mines with the promise of earning money to send to family and save for their marriage. However, often they are forced to work in the informal or artisanal sector, which comprises about 20% of the supply of cobalt. The informal sectors exploit adolescents with long hours and low pay (less than $2/day) and provide no safety or protective gear from the toxic dust in the mines [31]. Young girls that leave for the mine are not only forced to work long hours, they are also frequently exposed to sexual violence and exploitation to support themselves and send money home.

### Limitations

Our study has limitations. The study built off of the infrastructure of an existing randomized control trial (RCT) testing the effectiveness of the hybrid microfinance/asset transfer program, PFP, with adults in 10 rural villages. To successfully enroll young female and male adolescents (ages 10–15), our partners felt the trust of parents/guardians was essential in this challenging and remote humanitarian setting. The adolescents enrolled in RFR were not selected at random from the village households for participation, but were chosen with the support of parents already participating in PFP or interested in PFP that had an eligible adolescent living in the household. As a result, the study findings are not representative of all rural young adolescents, but instead young adolescents assenting to participate after endorsement and consent of parents. All data is self-report from young adolescents, therefore, the study’s self-reported measures may have been subject to response biases. The analyses are based on intention-to-treat principles which provides an unbiased but conservative estimate of the intervention effect possibly increasing the Type II error rate. Further, the girl and boy participants in the in-depth interviews were purposively selected by staff based on their participation in RFR with an effort to identify girls and boys that were successful and those that struggled in the program. Purposive sampling limits the ability to link our findings from the qualitative interviews to other participants in the study.

## Conclusions

This study underscores the potential for integrating economic empowerment programs with both parents and young adolescents to improve economic, education, and health outcomes for young adolescents. Increased asset building (i.e., resources that will move them towards economic well-being now and in the future), school attendance, and prosocial behaviors are clearly linked to parents/caregivers having the resources and ability to create and support an enabling environment to buffer the multiple and inter-related factors that increase vulnerability for boys and girls growing up in rural and complex humanitarian settings.

## Data Availability

The dataset analyzed during the current study is available by contacting the lead author.

## Abbreviations

ACEs: Adverse childhood events
APAI: Acholi Psychosocial Assessment Instrument
DRC: Democratic Republic of Congo
GEAS: Global Early Adolescent Study
HDDS: Household Dietary Diversity Scale
IPV: Intimate partner violence
IRB: Institutional Review Board
LMIC: Low- and middle-income countries
PAIDEK: Programme d’Appui aux Initiatives Economiques du Kivu
PFP: Pigs for Peace
PTSD: Post-traumatic stress disorder
RFR: Rabbits for Resilience
RRMP: Rapid Response to Movements of Populations
UNICEF: United Nations Children’s Fund
